# Estimation of hepatitis C prevalence in the Punjab province of Pakistan: A retrospective study on general population

**DOI:** 10.1371/journal.pone.0214435

**Published:** 2019-04-03

**Authors:** Asma Ahsan, Adnan Zafar Khan, Hasnain Javed, Shaper Mirza, Safee Ullah Chaudhary, Syed Shahzad-ul-Hussan

**Affiliations:** 1 Department of Biology, Syed Babar Ali School of Science and Engineering, Lahore University of Management Sciences, Lahore, Pakistan; 2 Punjab AIDS Control Program, Government of Punjab, Lahore, Pakistan; University 9 of July, BRAZIL

## Abstract

**Background:**

Hepatitis C virus (HCV) infections are amongst the leading public health concerns in Pakistan with a high disease burden. Despite the availability of effective antiviral treatments in the country the disease burden in general population has not lowered. This could be attributed to the asymptomatic nature of this infection that results in lack of diagnosis until the late symptomatic stage. To better estimate and map HCV infections in the country a population-based analysis is necessary for an effective control of the infection.

**Methods:**

Serologic samples of ~66,000 participants from all major cities of the Punjab province were tested for anti-HCV antibodies. The antibody-based seroprevalence was associated with socio-demographic variables including geographical region, age, gender and sex, and occupation.

**Results:**

Overall serological response to HCV surface antigens was observed in over 17% of the population. Two of the districts were identified with significantly high prevalence in general population. Analysis by occupation showed significantly high prevalence in farmers (over 40%) followed by jobless and retired individuals, laborers and transporters. A significant difference in seroprevalence was observed in different age groups amongst sex and genders (male, female and transgender) with highest response in individuals of over 40 years of age. Moreover, most of the tested IDUs showed positive response for anti-HCV antibody.

**Conclusion:**

This study represents a retrospective analysis of HCV infections in general population of the most populated province of Pakistan to identify socio-demographic groups at higher risk. Two geographical regions, Faisalabad and Okara districts, and an occupational group, farmers, were identified with significantly high HCV seroprevalence. These socio-demographic groups are the potential focused groups for follow-up studies on factors contributing to the high HCV prevalence in these groups towards orchestrating effective prevention, control and treatment.

## Introduction

Hepatitis C virus (HCV) infections progressively lead to liver impairment, cirrhosis and hepatocellular carcinoma [1]. Since the discovery of HCV in 1989, these infections continued to propagate across the globe despite extensive research to understand various aspects of the virus and the disease [2]. Currently, 71 million people are estimated to have chronic hepatitis C in the world, which are at the risk of developing liver hepatocellular carcinoma [3]. The global annual incident rate of HCV infections is over 3 millions [3–5] and mortality rate of its associated disorders stands at 0.4 million [6–8]. The prevalence of hepatitis C in Pakistan is possibly the second highest in the world with an estimated 10 million people (~5% of the population) affected [8–15]. Factors contributing to the high HCV infection rates in Pakistan include, unsafe practices of medical equipment by healthcare providers and dentists, unnecessary clinical use of injections, unhygienic state of instrumentation at barber salons, sharing of needles by drug users and unsafe blood transfusion [12, 15]. Additionally, lack of awareness amongst general population regarding factors associated with viral transmission is another underlying factor for the spread of the disease [16].

Several efficacious direct acting anti-HCV treatments have become available to general population in Pakistan as part of Government’s hepatitis control programs [17]. However, due to the asymptomatic nature of hepatitis C and lack of routine medical examinations, numerous HCV infected individuals with low-grade viremia remain unaware of their infection status for years and therefore, do not pursue treatment until the symptomatic stage of liver impairment [18]. These individuals then also contribute to the spread of the virus to general population. Such situation hampers the efforts for controlling the HCV infections even with the availability of effective treatments. Population based studies to identify specific socio-demographic groups with high HCV prevalence and an analysis of contributing factors is therefore, needed to control the disease in general population. In recent years several HCV related epidemiological studies have been conducted in Pakistan, which provide an overview of HCV prevalence. However, these investigations were limited to small population size [19, 20] or only to high- risk groups (IDUs, blood donors, health care workers) [21, 22] covering very small geographical regions [23].

In the current study, we have measured the seroprevalence of anti-HCV antibodies to perform retrospective analysis of HCV infections in general population of major cities of the Punjab province that accommodates 53% of the total population of Pakistan. Aim of this analysis was to identify socio-demographic groups with higher HCV prevalence so that these groups could be further investigated for factors contributing to higher HCV infections. Findings from the study will help in better management of hepatitis C prevention and treatment strategies in the country.

## Materials and methods

A total of 66,086 individuals participated in this study by visiting screening camps established for a week by Punjab AIDS Control Program (PACP) in year 2017, in 80 different towns across the Punjab province after a campaign was run through print media and by displaying posters at public places to encourage general public to test for HCV. Institutional review board of PACP and Lahore University of Management Sciences approved this study. No pre-selection criterion was applied for participation. For measuring serological response to HCV surface antigens, RAPID ICT test kit (One Step Hepatitis C Virus Test, Alere, Cat. No 02FK10) was used according to manufactures instruction. Informed consent was obtained from every participant before collecting samples. Each collected sample referred to a distinct individual as every individual was given a specific identification number (ID) that was related to their national ID card number ruling out the possibility of retesting. To ensure reproducibility and quality of the data approximately 5% of the samples were re-tested through ELISA to validate the results after collecting samples in EDTA vacutainer tubes. Participants’ socio-demographics were recorded, which included, age, sex and gender, occupation and city of residence. Indeed, complete information about socioeconomic data and characteristics of the regions (rural or urban) would have been much better. However, information about only four demographic variables was collected at this stage due to budget, time and staff limitations. Some socioeconomic information could be indirectly derived from this data. For example, the occupation category "farmers" means the participants were from rural area, and a "student" means the participant is in a school or college.

### Statistical analysis

Data was analyzed using IBM SPSS statistics version 23.0. Pearson’s chi square test was applied to determine correlation between categorical variables using significance threshold of P ≤ 0.05. Age as a continuous variable was categorized into groups of decades including <20, 21–30, 31–40, 41–50, 51–60, 61–70 and >70 years for each of the three genders, male, female and transgender. Univariate analysis was performed to determine the anti-HCV antibody prevalence in relation to any of the four independent variables including age, geographical region, sex and gender, and occupation. Subsequently, bivariate and multivariate analyses were performed while controlling the effect of potential confounders. Prevalence ratios (PR) with 95% confidence interval (95% CI) were also calculated as a measure of association [24]. For multivariate analysis, Fisher’s linear discriminant analysis was performed and the classification function coefficients were used to construct the membership function of age, occupation, and sex and gender. Each independent variable was converted into a categorical variables and a linear classifier for HCV status was constructed. Box’s model was employed for evaluating goodness of fit of the resulting model. Receiver operator characteristic curve was plotted to compare the sensitivity and specificity of the linear classification model. The predicted membership LDA functions for anti-HCV antibody response are given below:

Discriminantfunction(LDA)|NonReactive=−13.362+1.553(Occupation)+7.070(Gender)+3.520(Age_Groups)

Discriminantfunction(LDA)|Reactive=−14.248+1.453(Occupation)+7.189(Gender)+3.904(Age_Groups)

## Results

**Fig 1:**
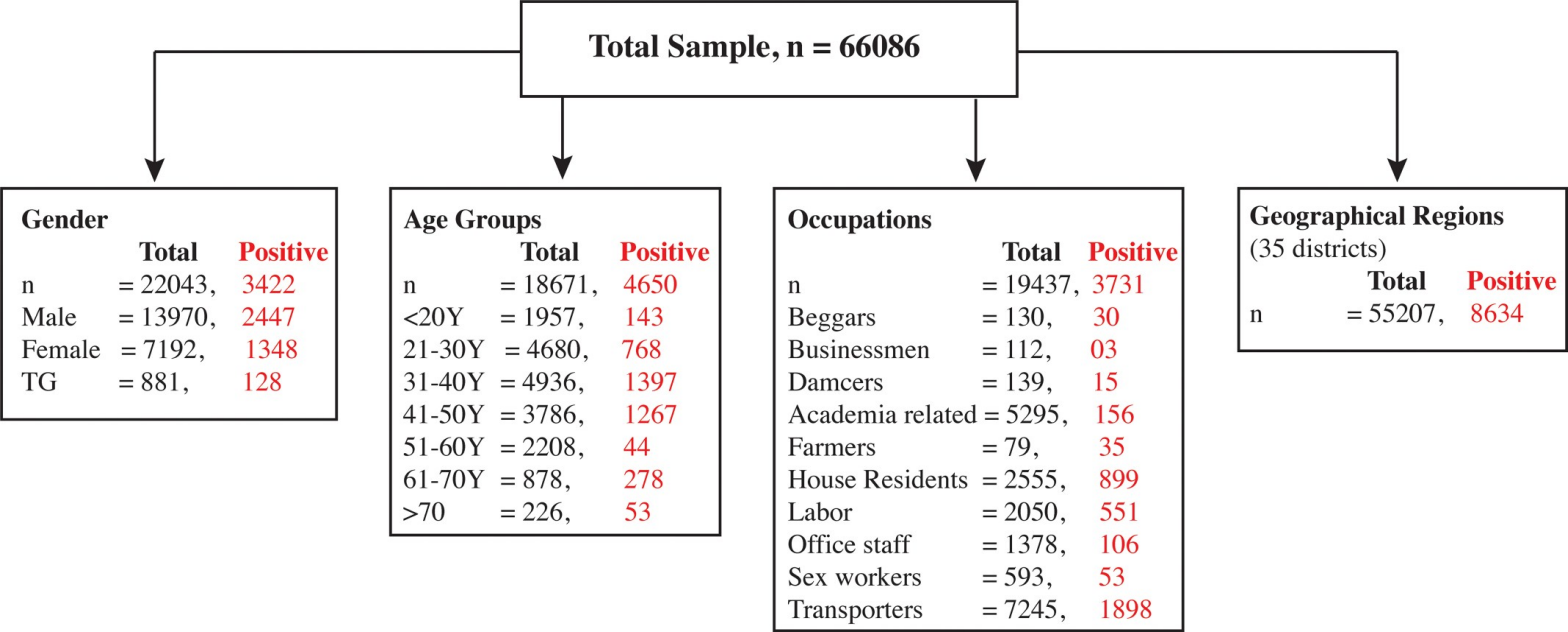
A summary of HCV seroprevalence in the Punjab province of Pakistan measured in year 2017.

Study was conducted on a total of 66,086 subjects. Out of the total participants, sex and gender information of 22043 individuals (33%), age information of 18671 individuals (28.1%), occupation information of 19437 individuals (29.3%) and resident city information of 55207 individuals (83.5%) was available (Fig 1).

**Fig 2:**
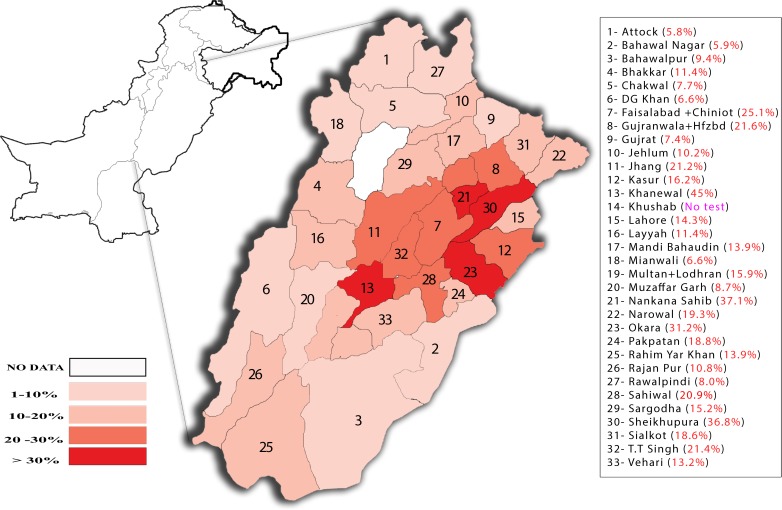
HCV seroprevalence in different geographical regions of the Punjab province in year 2017. Seroprevalence in percentage of the total tested samples in the respective regions is color-coded. Data from three districts, Chiniot, Hafizabad and Lodhran were combined with neighboring larger districts as mentioned in the legend. Due to unexpected logistical reasons, district Khushab could not be surveyed and therefore no data available from that district. The map was manually populated, using Adobe Illustrator software, with data obtained from descriptive analysis (percentage prevalence).

Overall 17.3% of the participants demonstrated a positive response for anti-HCV antibodies. In terms of geographical regions a sporadic distribution of seroprevalence was observed ranging from around 5% to 45% in different areas of the Punjab province. Five districts of Punjab including Khanewal (45%, *P*<0.01), Nankana Sahib (37.1%, *P*<0.01), Sheikhupura (36.8% (*P*<0.01), Okara (31.2%, *P*<0.01) and Faisalabad (25.1%, *P*<0.01) were identified with significantly high seroprevalence (Fig 2 and Table A in S1 File) as compared to other districts.

**Fig 3:**
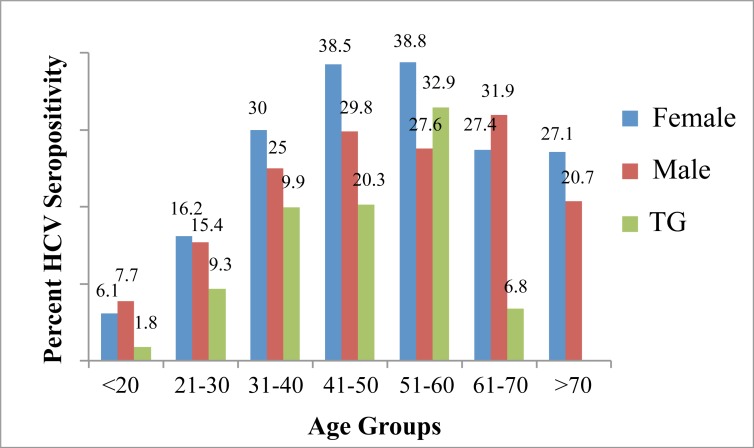
Sex and gender specific HCV seroprevalence in different age groups in the province of Punjab analyzed in year 2017.

While analyzing the seroprevalence in different age groups, a significantly high prevalence was observed within 41–60 years of age as over 33% (*P*<0.01) of the tested individuals in this age group were positive for anti-HCV antibodies (Table B in S1 File). In gender-based analysis, higher seroprevalence was observed in females as 18.7% (*P*<0.01) of the tested females demonstrated positive anti-HCV antibody response as compared to 17.5% and 14.5% in males and trans,s, respectively. In bivariate analysis by combining age, and sex and gender, the age group with the highest seroprevalence in females was observed to be 51–60 years (38.8%) [PR 1.73, 95% *CI* 1.486–2.024]. However, in males and transgenders the highest prevalence was observe in age groups 61–70 years (31%) [PR 1.64, 95% *CI* 1.340–2.015] and 51–60 years (32.9%) [PR 2.87, 95% *CI* 1.867–4.414], respectively (Fig 3, Table 1).

**Table 1:** HCV seroprevalence in different age groups of every sex and gender in the province of Punjab in year 2017. Prevalence ratios and *P*-values were calculated while comparing a specific age group with all other age groups of a specific sex and gender.

	Age	Total	Positive	% Positive	PR	*P*-value	95% CI
	<20	556	34	6.1	0.178	<0.001	0.127–0.250
	21–30	1036	168	16.2	0.530	<0.001	0.450–0.620
**Females (4674)**	31–40	1199	360	30.0	1.170	0.003	1.056–1.303
	41–50	973	375	38.5	1.71	<0.001	1.533–1.917
	51–60	577	224	38.8	1.73	<0.001	1.486–2.024
	61–70	263	72	27.4	1.03	0.820	0.792–1.341
	>70	70	19	27.1	0.94	0.940	0.604–1.718
	<20	1067	83	7.7	0.29	<0.001	0.235–0.366
	21–30	2892	444	15.4	0.64	<0.001	0.583–0.699
**Males (10706)**	31–40	2908	728	25.0	1.17	<0.001	1.094–1.260
	41–50	2087	622	29.8	1.49	<0.001	1.377–1.622
	51–60	1244	343	27.6	1.34	<0.001	1.193–1.504
	61–70	405	129	31.9	1.64	<0.001	1.340–2.015
	>70	111	23	20.7	0.92	0.700	0.582–4.451
	<20	110	2	1.8	0.11	0.090	0.543–1.523
	21–30	280	26	9.3	0.599	0.002	0.419–0.856
**TG (877)**	31–40	196	39	19.9	1.454	0.017	1.081–1.955
	41–50	158	32	20.3	1.486	0.026	1.058–2.087
	51–60	79	26	32.9	2.87	<0.001	1.867–4.414
	61–70	44	3	6.8	0.43	0.130	0.135–1.362
	>70	11	0	0			

To measure HCV prevalence with respect to occupation, on the basis of available data occupation was categorized into 10 categories including beggars, businessmen, dancers, academia-associated (students and staff members of schools, colleges and universities), farmers, house residents (housewives, and retired and jobless individuals), laborers (security guards, electricians, motor mechanics, shopkeepers, technicians, plumbers etc), office staff, sex workers and transporters (drivers, truckers, conductors, helpers and loaders). Four occupations were identified with significantly high anti-HCV antibody prevalence, which included farmers (44.3%, *P* <0.01) house residents (35.2%, *P* <0.01), laborer (26.9%, *P* <0.01) and transporter (26.2%, *P* <0.01) (Table 2). However, the participants related to academia such as students and faculty or those working in close association with academia, or individuals involved in trade or businessmen demonstrated significantly lower HCV prevalence (2.9% and 2.7%, respectively). In bivariate analysis, anti-HCV antibody prevalence in different occupation was analyzed while controlling the effect of age as potential confounder. Farmers and house resident groups demonstrated significantly high prevalence (S1 Fig).

**Table 2:** HCV seroprevalence with respect to different occupations in the province of Punjab, measured in year 2017.

Occupations	Total	Seropositive	% +ve	*P*-value	PR	95% CI
Beggars	130	30	23.1	0.260	1.263	0.841–1.896
Businessmen	112	3	2.7	<0.001	0.116	0.037–0.365
Dancers	139	15	10.8	0.012	0.511	0.300–0.872
Academia related	5295	156	2.94	<0.001	0.128	0.109–0.149
Farmers	79	35	44.3	<0.001	3.350	2.151–5.212
House residents	2555	899	35.2	<0.001	2.280	2.125–2.458
Laborers	2050	551	26.9	<0.001	1.550	1.413–1.695
Office staff	1378	106	7.7	<0.001	0.351	0.289–0.426
Sex workers	593	53	8.9	<0.001	0.413	0.312–0.547
Transporters	7245	1898	26.2	<0.001	1.490	1.44–1.55
Total	19437	3731	19.2			

**Fig 4:**
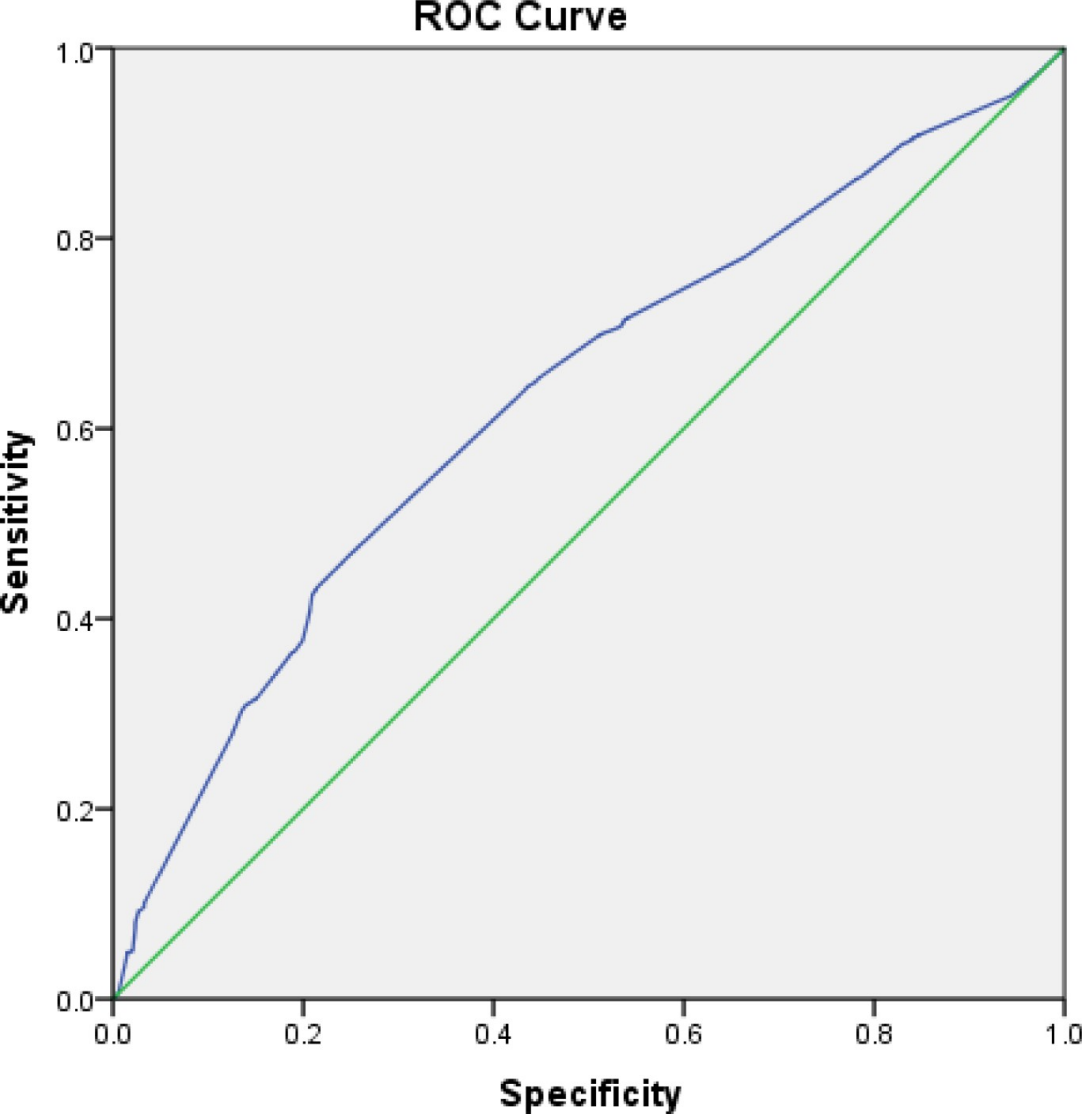
Receiver operator characteristic curve showing the true positive rate (sensitivity) vs. false positive rate (100-specificity) for the discriminant function for reactive and non-reactive HCV. The area under the curve (blue) i.e. the accuracy of the model is computed to be 0.634.

Fisher’s linear discriminant analysis revealed that age, occupation, and sex and gender all are strong determinants of HCV prevalence (Table 3). ROC plot showed that the classification rate between reactive and non-reactive HCV has 63.4% accuracy, which can provide high-confidence classification for further HCV prevalence studies in the province (Fig 4). In addition to the socio-demographic variables discussed above, 52.0 of the participants declared themselves as Injecting Drug Users (IDUs), 46.0 (88%) of those showed positive response for the presence of anti-HCV antibodies.

**Table 3:** Fisher's linear discriminant functions for classification of ‘Reactive’ and ‘Non-Reactive’ cases. The coefficients of the classification function include Age Group, Sex and Gender, and Occupation. The values for each of these coefficients have been tabulated separately for both ‘Non-Reactive’ (no HCV) and ‘Reactive’ (with HCV) cases.

Classification Function Coefficients		
	Response	
	Non-Reactive	Reactive
Age_Group	3.520	3.904
Sex and Gender	7.070	7.189
Occupation	1.553	1.453
(Constant)	-13.262	-14.248

## Discussion

Many epidemiological studies on HCV infections have been conducted in Pakistan in recent years. However, these studies show very inconsistent findings possibly because most of these studies are conducted by targeting small geographical region or by including only a specific population with very small sample size [19–21, 23, 25]. In the present study major geographical regions of the most populated province of Pakistan that accommodates 53% of the total Pakistani population, were covered incorporating a large sample size of population from 80 different cities and towns with diverse socio-demographic backgrounds involving all age groups, sex and genders, and association of these socio-demographic variables with HCV seoprevalence was analyzed.

In terms of geographical distribution, in some areas anti-HCV antibodies based serological response was observed in significantly high percentage of the regional population. In this regard five districts including Khanewal, Nankana Sahib, Sheikhupura, Okara and Faisalabad were identified with over 25% HCV seroprevalence. The analysis outcome in three of these regions, Khanewal, Nankana Sahib and Sheikhupura where number of participating individuals was very small (SI, Table 1), may not represent real situation due to a small sample size. Nevertheless, HCV prevalence in Okara and Faisalabad districts was significantly high where 2705 and 5734 individuals participated in the study, respectively and over 25% showed positive HCV serological response. The prevalence also varied with respect to age but in sex and gender specific way. The age group with highest HCV serological response was 51–60 years in females, 61–70 years in males and 51–60 years in transgenders. In previous studies male sex has been reported with higher HCV prevalence as compared to females in Pakistan (19). In this population-based study, significantly higher HCV prevalence was observed in females as compared to males. However, contributing factors in this regard remain to be determined. A remarkable variation was observed in HCV prevalence among different occupational groups. A significantly high HCV prevalence was observed among farmers (~44%). The major factor contributing to the high HCV prevalence among farmers could be the sharing of shaving razors among multiple individuals. In countryside and rural areas of Pakistan there has been a culture of one barber for a village under a contract of providing service for obtaining crops and grains from farmers. These barbers lacked any system of hygiene and generally used a special non-replaceable razor for possibly hundreds of times for different customers significantly contributing to the spread of diseases. It is also likely that these people are being given injections at the clinics of their primary healthcare without changing needles. Other participating groups with high HCV prevalence included house residents (house wives, jobless and retired individuals), laborer and transporters. Around 1/3^rd^ of the total participating individuals were truck/bus/taxi drivers and associated helpers and over 26% of these were positive for anti-HCV antibody response. Two occupational groups including students and staff of educational institutions, and businessmen showed a significantly low HCV seroprevalence (<3%) suggesting the awareness about the virus transmitting factors is possibly the main factor to prevent HCV infection. Interestingly the sex workers, which were considered as a high-risk group, did not show high prevalence as hepatitis C has not been declared as sexually transmitted disease.

In this study, a special emphasis was given to include socially underprivileged transgender community, to determine HCV prevalence through the specific efforts of the staff of screening camps. Access to proper health care for a large proportion of the Pakistani population is limited and this situation is further exacerbated for transgenders. Transgender communities in South Asia are socially excluded and face disparities in healthcare, education, career opportunities and in acquisition of proper status in the society [26]. As a result a vast majority of these individuals become commercial sex workers or beggars to make its financial needs [27]. In Pakistan incident rates of HIV infections among transgenders are very high where 17.5% of the total reported HIV infected cases are transgenders [28]. In order to find if similar situation exists for HCV infections, available data of 877 transgenders from the screening camps were analyzed and found HCV prevalence in these individuals is not higher than their counterpart cis genders. High rate of HIV infections among transgenders is attributed to sexual transmissions, as majority of the transgenders works as sex workers. Exposure to HCV risk factors for transgenders is possibly the same as that for general population. Another faction of the society, that faces health disparities in Pakistan are IDUs. Most of the IDUs belong to lower middle class families and most of these people are jobless. An effective rehabilitation program does not exist for such individuals. In this study over 80% of the tested IDUs were found positive for anti-HCV antibodies. Although the sample size was not large enough, only 43 participants, to draw a conclusion, yet high percentage of the prevalence among tested individuals is indicative of seriousness of the situation and urges to perform a large scale study.

World Health Organization and some other studies report HCV incident rates as 4.5% in Pakistan [29–31] while many recent studies report up to 15% HCV prevalence [11, 19, 32] corroborating our current analysis that suggests HCV seroprevalence as 17% in the major part of Pakistan. The current study is based on the presence of anti-HCV antibody in the serum, which does not necessarily represent the situation analysis of active viremia. However, this study represents the retrospective analysis of HCV infections irrespective of the presence of active viremia or treatment-induced or spontaneous clearance of the virus and highlights that certain socio-demographic groups have significantly high exposure to HCV infections [33–35]. This high exposure may not be directly related to the specific demographic background such as being farmer or truck driver or laborer, but behavior, such as using injectable drugs, and lack of awareness common in certain socio-demographic background may be the direct risk factors. Moreover, in these identified socio-demographic groups detailed investigation on risk factors such as unnecessary clinical use of injections, unsafe practices of medical equipment by dentists, unsafe blood transfusion, reuse of shaving razors and unhygienic state of instrumentation at barber salons needs to be done in the follow up studies. Despite the limitations regarding availability of data on all selected socio-demographic variables from all participants resulted from unwillingness of a proportion of the participants to provide complete demographic information, the current study identifies certain socio-demographic groups with high HCV prevalence.

In conclusion, we performed retrospective study of HCV infections by analyzing the prevalence of anti-HCV antibodies involving over 60,000 participants from all major cities of the Punjab and the key findings are highlighted as follow: socio-demographic variables such as age, sex and gender, and occupation were associated with HCV seroprevalence. Females demonstrated significantly higher HCV prevalence as compared to males and transgenders. Farmers were identified with the highest HCV prevalence among all occupational groups, though jobless people, transporters and laborers also demonstrated significantly high prevalence. In terms of geographical regions, HCV prevalence in two of the districts of Punjab, Faisalabad and Okara was found significantly high in general population that urges detailed analysis of risk factors in those areas and proper measures to control these infections. Moreover, considering the magnitude of hepatitis C as public health problem in Pakistan, health authorities are urged to enhance their focus on following measures: (i) develop prevention campaigns, especially targeted at the most affected groups, (ii) intervene in establishments that are known to contribute to maintaining the high prevalence of Hepatitis C in order to guide and monitor safe procedures and penalize those establishments that do not follow such guidelines, (iii) provide access and monitor adherence to treatment indicated in these cases, (iv) develop and implement an official government information system for reporting hepatitis C positive cases diagnosed in routine health services, making this disease as compulsory notification, and (v) periodically prepare epidemiological reports on the disease in order to follow the behavior of this epidemic, in addition to seeking to improve the quality and completeness of the data in order to have a better characterization of the disease and regional differences observed.

## Supporting information

S1 Fig Anti-HCV antibody seroprevalence in different occupational groups while controlling the age as confounder. Anti-HCV antibody positive population is shown in percent of the total population in every group.
(PDF)

S1 File Seroprevalence of anti-HCV antibodies in 32 districts of the Punjab province (Table A). Anti-HCV antibody seroprevalence in different age groups of the total tested samples (Table B). Anti-HCV antibody seroprevalence in different genders (Table C).
(DOCX)
