# Supplementary figures and images for: Estimation of hepatitis C prevalence in the Punjab province of Pakistan: A retrospective study on general population
Source: PLoS One. 2019 Apr 3;14(4):e0214435. doi: 10.1371/journal.pone.0214435 (PMC6447227; doi:10.1371/journal.pone.0214435)

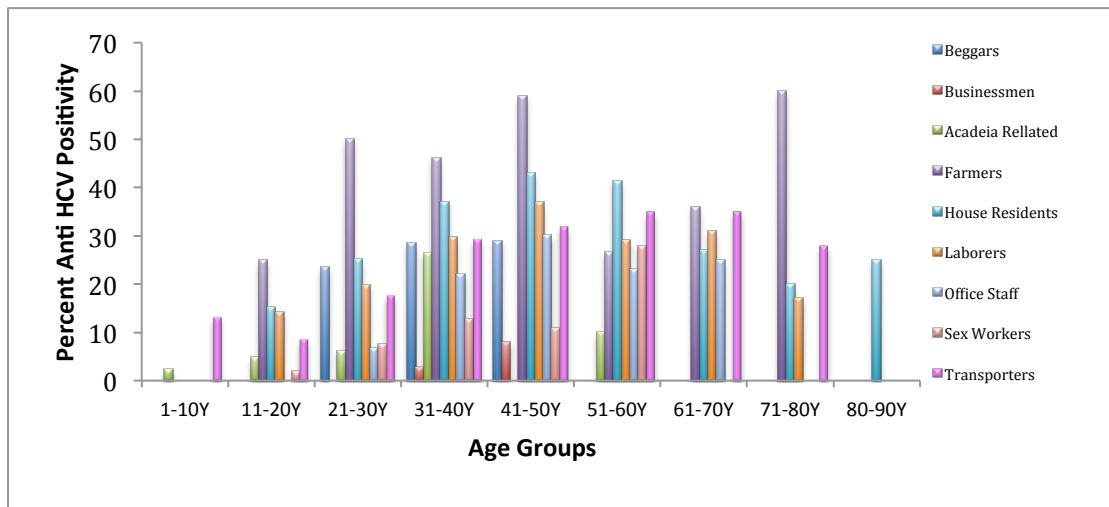

Supplement: S1 Fig — Anti-HCV antibody positive population is shown in percent of the total population in every group. (PDF) [file pone.0214435.s001.pdf]
