# Supplementary material for: Estimation of hepatitis C prevalence in the Punjab province of Pakistan: A retrospective study on general population
Source: PLoS One. 2019 Apr 3;14(4):e0214435. doi: 10.1371/journal.pone.0214435 (PMC6447227; doi:10.1371/journal.pone.0214435)
Supplement: S1 File — Seroprevalence of anti-HCV antibodies in 32 districts of the Punjab province (Table A). Anti-HCV antibody seroprevalence in different age groups of the total tested samples (Table B). Anti-HCV antibody seroprevalence in different genders (Table C). (DOCX) [file pone.0214435.s002.docx]

|  | **City** | **Total** | **Seropositive** | **% Positive** | ***P*-value** |
| --- | --- | --- | --- | --- | --- |
| 1 | Attock | 2084 | 121 | 5.8 | <0.001 |
| 2 | Bahawal Nagar | 68 | 4 | 5.9 | 0.027 |
| 3 | Bahawalpur | 3392 | 320 | 9.4 | <0.001 |
| 4 | Bhakkar | 245 | 28 | 11.4 | 0.069 |
| 5 | Chakwal | 181 | 14 | 7.7 | 0.003 |
| 6 | DG Khan | 828 | 55 | 6.6 | <0.001 |
| 7 | Faisalabad | 5734 | 1440 | 25.1 | <0.001 |
| 8 | Gujranwala | 3247 | 703 | 21.6 | <0.001 |
| 9 | Gujrat | 1209 | 211 | 17.4 | 0.079 |
| 10 | Jehlum | 776 | 79 | 10.2 | <0.001 |
| 11 | Jhang | 1574 | 335 | 21.2 | <0.0010 |
| 12 | Kasur | 1144 | 186 | 16.2 | 0.56 |
| 13 | Khanewal | 20 | 9 | 45 | <0.001 |
| 14 | Lahore | 8040 | 1148 | 14.3 | <0.001 |
| 15 | Layyah | 245 | 28 | 11.4 | 0.069 |
| 16 | Mandi Bahauddin | 537 | 75 | 13.9 | 0.284 |
| 17 | Mianwali | 3314 | 220 | 6.6 | <0.001 |
| 18 | Multan | 3622 | 577 | 15.9 | 0.6 |
| 19 | Muzaffar Garh | 460 | 40 | 8.7 | <0.001 |
| 20 | Nankana Sahib | 35 | 13 | 37.1 | <0.001 |
| 21 | Narowal | 31 | 6 | 19.3 | 0.56 |
| 22 | Okara | 2705 | 845 | 31.2 | <0.001 |
| 23 | Pakpattan | 526 | 99 | 18.8 | 0.44 |
| 24 | Rahim Yar Khan | 3023 | 421 | 13.9 | 0.008 |
| 25 | Rajan Pur | 231 | 25 | 10.8 | 0.043 |
| 26 | Rawalpindi | 5644 | 450 | 7.97 | <0.001 |
| 27 | Sahiwal | 1795 | 375 | 20.89 | <0.001 |
| 28 | Sargodha | 1958 | 297 | 15.2 | 0.56 |
| 29 | Sheikhupura | 114 | 42 | 36.8 | <0.001 |
| 30 | Sialkot | 1733 | 323 | 18.6 | 0.1 |
| 31 | T.T.Singh | 654 | 140 | 21.4 | <0.001 |
| 32 | Vehari | 38 | 5 | 13.2 | 0.67 |
|  |  | 55207 | 8634 |  |  |

**Table A in S1 File**. **Seroprevalence of anti-HCV antibodies in 32 districts of the Punjab province**

**Table B in S1 File.** **Anti-HCV antibody seroprevalence in different age groups of the total tested samples**

| **Age groups** | **Total** | **Seropositive** | **% +ve** | **PR** | ***P*-value** | **95% CI** |
| --- | --- | --- | --- | --- | --- | --- |
| <20 Y | 1957 | 143 | 7.3% | 0.238 | <0.001 | 0.201-0.281 |
| 21-30 Y | 4680 | 768 | 16.4% | 0.592 | 0.001 | 0.552-0.635 |
| 31-40 Y | 4936 | 1397 | 28.3% | **1.190** | <0.001 | 1.130-1.254 |
| 41-50 Y | 3786 | 1267 | 33.5% | **1.517** | <0.001 | 1.430-1.608 |
| 51-60 Y | 2208 | 744 | 33.7% | **1.532** | <0.001 | 1.412-1.663 |
| 61-70 Y | 878 | 278 | 31.7% | **1.397** | <0.001 | 1.217-1.604 |
| 71-100 Y | 226 | 53 | 23.5% | 0.9 | 0.6 | 0.6-1.2 |
|  | **18671** | **4650** |  |  |  |  |

**Table C in S1 File. Anti-HCV antibody seroprevalence in different genders**

|  | **Total** | **Seropositive** | **% +ve** | **PR** | ***P*-value** | **95% CI** |
| --- | --- | --- | --- | --- | --- | --- |
| **M** | 13970 | 2447 | 17.5% | 0.981 | 0.151 | 0.955-1.007 |
| **F** | 7192 | 1348 | 18.7% | 1.065 | 0.011 | 1.015-1.118 |
| **TG** | 881 | 128 | 14.5% | 0.785 | 0.01 | 0.65-0.94 |
